# Tenecteplase—What Have We Learned till Now? A Narrative Review

**DOI:** 10.3390/life15091356

**Published:** 2025-08-27

**Authors:** Matija Zupan, Lara Straus, Pawel Kermer, Senta Frol

**Affiliations:** 1 Department of Vascular Neurology, University Medical Centre Ljubljana, 1000 Ljubljana, Slovenia; matija.zupan@kclj.si (M.Z.); lara.straus@kclj.si (L.S.); 2 Faculty of Medicine, University of Ljubljana, 1000 Ljubljana, Slovenia; 3 Department of Neurology, Nordwest-Krankenhaus Sanderbusch, Friesland Kliniken GmbH, 26452 Sande, Germany; pkermer@gwdg.de; 4 Department of Neurology, University Medical Center Göttingen, 37073 Göttingen, Germany

**Keywords:** tenecteplase, acute ischemic stroke, treatment, narrative review, alteplase

## Abstract

Tenecteplase (TNK), a genetically modified tissue plasminogen activator, has emerged as a promising alternative to alteplase (ALT) for intravenous thrombolysis (IVT) in acute ischemic stroke (AIS). Our aim was to synthesize the current clinical evidence on TNK use in AIS patients, comparing efficacy, safety, and workflow benefits to ALT. A narrative review was conducted by searching PubMed and Scopus (January 2024–March 2025) for studies comparing TNK and ALT in AIS. A total of 35 eligible papers were included. Data were grouped by treatment scenario: IVT-only, bridging before endovascular therapy (EVT), and intra-arterial thrombolysis (IAT). The results showed that TNK is non-inferior—and in some analyses, superior—to ALT regarding functional outcomes, especially in large vessel occlusion. TNK facilitates shorter treatment delays due to its single-bolus administration. While some trials report higher rates of intracranial hemorrhage, overall safety and mortality are comparable. In conclusion, TNK appears to exert equivalent or superior efficacy and safety compared to ALT in multiple AIS scenarios. Its pharmacological and logistical advantages support its broader clinical adoption. Further trials are needed, especially for IAT, central retinal artery occlusion, and patients on dabigatran.

## 1. Introduction

Intravenous thrombolysis (IVT) for acute ischemic stroke (AIS) significantly improves independent recovery in eligible patients [1,2]. For almost three decades, the standard thrombolytic agent has been alteplase (ALT) (0.9 mg/kg, to a maximum of 90 mg; 10% bolus, 90% in a 1 h infusion). Tenecteplase (TNK) is a modified recombinant tissue plasminogen activator in which three amino acid substitutions confer greater fibrin specificity, resistance to inactivation by plasminogen activator inhibitor 1, and a longer half-life [3]. Contrary to ALT, these features enable single-bolus administration of TNK (a single bolus of 0.25 mg/kg, to a maximum of 25 mg). This allows not only for substantial workflow advantages in endovascular therapy (EVT) but also for improvement in door-to-needle time (DNT), door-to-groin puncture time (DGPT) in EVT patients, and door-in-door-out time (DIDO) in EVT patient transfers [4]. What is more, the risks of underdosing due to infusion interruption or delayed infusion initiation after bolus can be avoided with TNK [5].

The body of evidence in favor of TNK use in AIS has been growing rapidly in recent years. Numerous randomized controlled trials (RCTs) within 4.5 h of last known well (LKW) have demonstrated the non-inferiority of TNK in comparison to ALT for AIS patients, with comparable efficacy and safety [6,7,8,9,10]. A meta-analysis of three RCTs, which also investigated TNK 0.25 mg/kg in the extended 24 h time window (ROSE-TNK, TWIST, and TIMELESS), demonstrated a favorable safety and efficacy profile for TNK, but ongoing RCTs are crucial for strengthening this evidence [11]. Collectively, available evidence suggests that TNK can be considered preferentially for IVT in AIS within 4.5 h of symptom onset.

In this narrative review, we summarize the available evidence and discuss the relevance and importance of TNK treatment in AIS patients in everyday clinical practice. In addition, we also discuss unresolved issues on TNK use in dabigatran-treated patients with AIS, patients with central retinal artery occlusion (CRAO) and off-label intra-arterial thrombolysis (IAT).

## 2. Materials and Methods

We searched PubMed and Scopus from the approval of TNK in Europe at the beginning of 2024 through 1 March 2025 for studies reporting TNK use compared to ALT in AIS patients. In addition, we searched references of related letters, reviews, and editorials to identify other potentially eligible studies. The search query included the following keywords: acute ischemic stroke, tenecteplase, alteplase, recombinant tissue plasminogen activator, endovascular treatment. To be eligible for the present narrative review, the studies had to be published full-text articles in English language. We systematically screened for the following data: efficacy/effectiveness (90-day functional outcome), safety (symptomatic intracranial hemorrhage—sICH, 90-day mortality), convenience of use, and cost. We divided the body of evidence into the following relevant clinical scenarios: TNK vs. ALT used in IVT as a sole treatment, TNK vs. ALT in patients before EVT (“bridging therapy”), and TNK vs. ALT in IAT as an adjunct treatment following EVT.

## 3. Results

Our literature search retrieved 55 papers, of which 35 were deemed relevant. In total, we included 11 RCTs, nine meta-analyses, 12 real-world studies (RWSs)/case series (CSs), and three opinions/reviews/editorials.

### 3.1. Clinical Scenario 1: Tenecteplase vs. Alteplase for Intravenous Thrombolysis as a Sole Reperfusion Strategy in Acute Ischemic Stroke Patients

To assess available evidence for this clinical scenario, we included ten RCTs, seven meta-analyses, six RWSs/CSs, and three reviews/opinions/editorials.

#### 3.1.1. Randomized Controlled Trials

Parsons et al. conducted the TASTE trial, a multicenter, randomized, controlled, phase 3 non-inferiority study including 680 patients (TNK 339 and ALT 341). In the “per-protocol” population (295 TNK, 306 ALT), TNK was shown to be non-inferior to ALT in terms of functional outcomes, although superiority was not reached. No significant differences were observed in all-cause mortality or sICH between the treatment groups. Additionally, the study highlighted the utility of CT perfusion imaging in guiding IVT, even within the standard therapeutic time window [6].

The ATTEST-2 trial, performed in the UK, evaluated non-inferiority or superiority of TNK versus ALT within 4.5 h of symptom onset and included 1777 patients who received IVT (885 TNK and 892 ALT). TNK was non-inferior, but not superior, to ALT for the modified Rankin Score (mRS) at 90 days. No significant differences in all-cause mortality and sICH were observed. The study also highlighted the easier administration of TNK, especially in the context of interhospital transfers [12].

The TEMPO-2 trial was a randomized, open-label, phase 3 superiority trial including patients with minor AIS (National Institute of Health Stroke Scale (NIHSS) 0–5) and a confirmed intracranial occlusion, of whom 11% had large vessel occlusion (LVO), and 54% had medium vessel occlusion (MeVO) or focal perfusion abnormality within 12 h of onset. TNK (0.25 mg/kg) was compared to dual antiplatelets (DAPTs), but not with ALT. A total of 886 patients were enrolled (454 DAPT, 432 TNK). There were no significant differences in the primary outcome (return to premorbid mRS) or in sICH rates. However, mortality was significantly higher in the TNK arm compared with DAPT, leading to an early termination of the trial for futility [13].

The TRACE-2 study assessed the efficacy and safety of 0.25 mg/kg TNK compared to 0.9 mg/kg ALT in AIS patients aged ≥80 years in China. Among the 137 participants (75 TNK, 59 ALT), functional outcomes (mRS 0–1 at 90 days) and sICH rates did not differ significantly. Notably, the median cost for 0.25 mg/kg TNK IVT was around 30% lower than that for 0.90 mg/kg ALT [14].

Liu et al. investigated the efficacy and safety of TNK versus ALT in 1382 patients (688 TNK, ALT 694), stratified by diabetes status and admission hyperglycemia. The primary efficacy outcome (mRS 0–1 at 90 days) and risk of sICH were comparable between the treatment groups, regardless of hyperglycemia [15].

A study by Cimflova et al. included 136 patients (77 ALT, 59 TNK) with posterior circulation vessel occlusion, of whom 28 underwent EVT. No significant differences were observed between TNK and ALT in terms of 90-day mRS 0–1, sICH, mortality, or successful reperfusion (extended thrombolysis in cerebral infarction—TICI ≥ 2b/3) [16].

Bala et al. conducted a study in 128 patients with ≥70% extracranial internal carotid artery (ICA) stenosis and concurrent intracranial ICA or M1/M2 occlusions. Among the 35 patients who received IVT without EVT, no significant outcome differences were observed between TNK and ALT [17].

In another study by Bala et al., involving 1558 AIS patients, 455 were identified with MeVO. Of these, 368 received IVT without EVT (189 TNK and 179 ALT). The primary outcome (90-day mRS 0–1) did not differ significantly between groups (37.9% with TNK vs. 34.7% with ALT). The 90-day rates of sICH and mortality were similar in both groups. However, no subgroup analysis was performed for this IVT-only population [18].

Nair et al. evaluated 378 patients with minor AIS (NIHSS ≤ 5), comparing outcomes between TNK (*n* = 194) and ALT (*n* = 184). Safety (sICH, mortality) and efficacy (mRS 0–1) outcomes did not differ significantly between groups [19].

Cheng et al. studied 224 patients with AIS presenting 4.5 to 24 h after LKW, with favorable penumbral profiles and LVO or MeVO. Of these, 111 patients received TNK and 113 received the best medical treatment, including 26 patients treated with ALT. Outcome comparisons between TNK and ALT in this subgroup were not clearly defined [20].

#### 3.1.2. Meta-Analyses

Huang et al. conducted a meta-analysis of ten RCTs including 5123 patients (2677 TNK, 2446 ALT). No significant differences were observed between TNK and ALT in achieving excellent functional outcomes (mRS 0–1 at 90 days) or in the rate of sICH. Subgroup analysis, however, suggested that the 0.25 mg/kg dose of TNK was associated with greater efficacy and a lower rate of sICH compared with the 0.40 mg/kg dose of TNK [21].

Singh et al. analyzed data from nine studies involving 3573 patients and found no significant differences between TNK and ALT in terms of excellent functional outcomes (mRS 0–1) or all-cause mortality at 90 days. However, the incidence of intracranial hemorrhages (both symptomatic and asymptomatic) was higher in the TNK group [22].

Ma et al. performed a comprehensive meta-analysis including data from 12 randomized trials (*n* = 5533) and 24 non-randomized studies (*n* = 44,956). TNK and ALT showed no significant difference in achieving excellent functional outcomes at three months, but the 0.25 mg/kg dose of TNK was associated with improved outcomes. The rate of sICH was similar between the groups, while all-cause mortality was lower in the TNK group [23].

Xiong et al. analyzed data from phase III clinical trials involving 4068 AIS patients treated within the 4.5 h window (2056 TNK; 2012 ALT). TNK was found to be non-inferior to ALT in achieving excellent functional outcomes (mRS 0–1) and was not associated with increased risks of sICH or mortality [24].

Wang et al. conducted a meta-analysis of nine RCTs and reported no significant difference in the incidence of sICH (TNK: 48/1807 vs. ALT 44/1760) and mortality (TNK: 194/1743 vs. ALT 190/1704). However, TNK was associated with a significantly higher rate of excellent functional outcomes (TNK: 871/1798 vs. ALT: 792/1750) [25].

Koh et al. investigated ethnicity-specific differences in safety and efficacy of TNK compared to ALT. A significantly higher rate of excellent functional outcomes was observed in patients treated with TNK compared with ALT, particularly in Caucasians. No significant differences in mortality or sICH were noted between TNK and ALT across ethnic subgroups. In Asians, TNK was associated with a higher rate of complete recanalization compared with ALT [26].

Günkan et al. conducted a meta-analysis evaluating the safety and efficacy of IVT beyond the conventional 4.5 h window. No significant differences were found between TNK and ALT in terms of excellent functional outcomes, sICH, or mortality [27].

#### 3.1.3. Real-World Studies and Case Series

Cassano et al. included 48 AIS patients (24 TNK, 24 ALT) in a U.S.-based study. No differences were found between TNK and ALT regarding safety outcomes (mortality or sICH). While TNK in the U.S. appeared more cost-effective at face value (approximately USD 1100 cheaper per 50 mg kit compared with a 100 mg ALT vial), total expenditures with TNK may be higher due to drug waste [28].

Lo et al. conducted a retrospective review in Hong Kong involving 286 patients with suspected LVO, who were treated with either TNK (*n* = 148) or ALT (*n* = 138), without undergoing EVT. No significant differences were found between the two groups in terms of sICH, functional independence (mRS 0–2), or mortality [29].

Dutta et al. carried out a real-world, retrospective observational study with 154 AIS patients (71 TNK, 83 ALT), of whom seven underwent EVT. There were no significant differences in mRS 0–1 at 90 days, sICH rates, or mortality between the treatment groups [30].

A Chilean study (Guzman et al., 2025) evaluated 221 AIS patients: 110 received TNK during the COVID-19 pandemic, while 111 received ALT just prior to the pandemic. Among them, 39 underwent EVT. The safety and functional outcomes were similar between the TNK and ALT groups [31].

Skärlund et al. conducted a nationwide Swedish stroke registry study involving 7448 AIS patients (888 TNK, 6560 ALT), including 1277 who received EVT. TNK was found to be non-inferior to ALT regarding sICH, in-hospital and 90-day mortality, as well as functional outcomes [32].

Henderson et al. performed a single-center study involving 100 AIS patients (50 TNK, 50 ALT), with 21 patients undergoing thrombectomy. The study found no statistically significant differences between TNK and ALT in secondary safety or efficacy outcomes, yet there was a significant decrease in DNT with TNK compared with ALT [33].

#### 3.1.4. Review/Commentary/Editorial/Opinion

According to a commentary by Toyoda, convincing data from meta-analyses showed the superiority of TNK to ALT for an excellent outcome, which will soon shape the future AIS guidelines globally [34].

In their review, Campbell pointed out that TNK has been compared to ALT in more patients than ALT was historically compared to a placebo. Moreover, they highlight the superiority of TNK over ALT for an excellent 90-day outcome. The absolute benefit of TNK over ALT in the general IVT-eligible population at a mRS 0–1 threshold is clinically meaningful at ~3% (number needed to treat to achieve no disability of ~33). The benefit may be greater in patients with LVO. The simplicity of bolus administration (compared with 60 min ALT infusion) ensures the full dose is given and facilitates transport between or within hospitals for EVT. On the contrary, in patients with mild AIS (NIHSS < 5), TNK was associated with no benefit in functional outcomes compared to DAPT loading, despite increased recanalization with TNK, and there was an increase in sICH and mortality. The latest data using TNK have extended the time window to 24 h using perfusion-imaging selection. In patients with LVO without access to EVT, TNK vs. standard care demonstrated an absolute benefit in mRS 0–1 of ~10%, similar to ALT 0–3 h in the broad IVT-eligible population or 4.5–9 h in the perfusion mismatch population [35].

In their mini-review, Wang et al., on the basis of two studies, argue that in patients with minor non-disabling mild AIS, treatment with IVT (either ALT or TNK) vs. DAPT did not increase the likelihood of favorable 90-day functional outcomes [36].

### 3.2. Clinical Scenario 1: Summary of Findings

In eight out of ten RCTs, the functional outcomes, sICH frequency and mortality after IVT with TNK were comparable to IVT with ALT, even in the presence of diabetes and/or hyperglycemia, in patients aged ≥80 years, in posterior circulation vessel occlusion, in carotid tandem lesion, and in minor AIS [14,15,16,17,19]. Additionally, TNK use in China was associated with around 30% lower cost [14]. Interestingly, in patients with non-disabling minor AIS and proven intracranial occlusion, TNK was associated with higher mortality than the comparator (DAPT) [13]. In two out of seven meta-analyses, including more than 127,000 patients in total, TNK achieved better functional outcomes compared with ALT, especially in Caucasians, with more frequent complete recanalization in Asians [25,26]. In one meta-analysis, TNK was associated with a higher frequency of both sICH and asymptomatic intracranial hemorrhage, without a concomitant rise in mortality or worsening of functional outcomes [22]. Mortality was lower after TNK compared with ALT in one meta-analysis [23]. In all six included RWSs/CSs, there were no differences in the mortality and sICH occurrence between TNK and ALT. Yet, according to one study, TNK was associated with cost savings, although poor waste management could undermine its overall cost-effectiveness. In another study, the use of TNK instead of ALT resulted in a significantly shorter DNT, without any difference in functional outcomes and mortality [28,33]. Two out of three reviews emphasize the superiority of TNK over ALT for excellent functional outcomes, with one predicting that TNK will soon be introduced into AIS guidelines globally [34]. One of the reviews underlines the simplicity and ease of bolus administration of TNK, ensuring full-dose administration, with a likely greater benefit in LVO [35]. According to one review, the treatment time window with TNK is suggested to extend up to 24 h using perfusion-imaging selection [35]. The summary of findings for this clinical scenario are presented in Table 1.

### 3.3. Clinical Scenario 2: Tenecteplase vs. Alteplase Before Endovascular Treatment (“Bridging Therapy”)

To assess available evidence for this clinical scenario, we included four RCTs (all being sub-studies of the AcT study), two meta-analyses, six RWSs/CSs, and one review/opinion.

#### 3.3.1. Randomized Controlled Trials

In a study by Cimflova et al., 28 out of 1577 enrolled patients underwent EVT, with 16 receiving ALT and 12 receiving TNK. The results demonstrated no significant differences between the two groups in terms of favorable clinical outcomes (mRS 0–1 at 90 days), occurrence of sICH, baseline or final reperfusion rates, or mortality [16].

Bala et al. conducted a study involving 1577 patients, among whom 128 had carotid tandem lesions. A subgroup of 93 patients underwent EVT following IVT; 50 received TNK and 43 received ALT. In this cohort, TNK was associated with higher odds of achieving mRS 0–1 at 90 days after multivariable adjustment. However, no significant differences were observed between the groups in terms of successful intracranial recanalization, rates of sICH, or mortality [17].

In another study by Bala et al., involving 1558 patients, 455 were identified with MeVO. EVT was performed in 87 patients (47 TNK, 40 ALT). While the rates of 90-day mRS 0–2, sICH, and mortality were comparable between the two groups, the TNK group demonstrated significantly higher rates of final successful and excellent reperfusion [18].

A 2025 study by Bala et al. included 435 patients, with 222 receiving TNK and 213 treated with ALT prior to EVT. Although no significant differences in clinical efficacy or safety outcomes were observed between the groups for first-line EVT overall, TNK was associated with significantly higher odds of achieving final major reperfusion when the aspiration technique was employed. This effect was not observed when using stentriever devices [37].

#### 3.3.2. Meta-Analyses

A meta-analysis by Wu et al. included ten studies—comprising two RCTs and eight non-randomized non-blind cohort studies—encompassing a total of 3722 patients who underwent EVT due to AIS in either the anterior or posterior circulation (1266 received TNK and 2456 ALT). Treatment with TNK was associated with significantly higher early recanalization rate and lower mortality within 90 days compared with ALT. However, the rates of sICH and functional independence (defined as NIHSS 0–2) were comparable between the two groups [38].

A report by Guo et al. included eight RCTs with a total of 2836 AIS patients, who were divided into five subgroups based on the treatment received: EVT alone, 0.25 mg/kg TNK + EVT, 0.40 mg/kg TNK + EVT, 0.6 mg/kg ALT + EVT, and 0.9 mg/kg ALT + EVT. When comparing TNK and ALT, the lower dose of TNK was associated with a significantly higher rate of successful reperfusion (TICI 2b-3) compared with any dose of ALT. No significant differences were observed between groups in terms of mortality, functional outcomes, and sICH [39].

#### 3.3.3. Real-World Studies and Case Series

Hendrix et al. collected data on 635 patients with AIS and LVO from Pennsylvania, United States, who received IVT prior to EVT; 309 patients were treated with TNK and 326 with ALT. The study reported no significant differences between the two groups in terms of favorable functional outcomes (90-day mRS ≤2), successful endovascular reperfusion (modified TICI 2b–3), sICH, or 90-day all-cause mortality [40].

Hendrix et al. conducted another retrospective study involving 462 patients with LVO AIS who received IVT prior to EVT. The study aimed to investigate the association between admission hyperglycemia and sICH in patients treated with TNK (*n* = 254) versus ALT (*n* = 208). Among hyperglycemic patients, the incidence of sICH was comparable between TNK and ALT groups. Similarly, in normoglycemic patients, no significant differences in sICH rates were observed between the two thrombolytic agents [41].

Karamchandani et al. analyzed data from 233 patients with LVO AIS (109 TNK, 124 ALT), and 82% (191) received subsequent EVT (ALT 100, TNK 91). There were no significant differences in recanalization rates (for initial basilar, ICA, or middle cerebral artery (MCA)-M1 occlusion, modified TICI 2b-3 revascularization on initial angiogram; for EVT patients with initial MCA-M2 occlusion, modified TICI 2c-3 revascularization or absence of retrievable thrombus on initial angiogram), functional outcomes (mRS 0–2), or safety (sICH) between the treatment groups in each of these subsets of patients [42].

A French study by Zarzour et al. evaluated 1131 patients who underwent EVT, including 250 treated with TNK and 881 with ALT. Functional independence at 90 days (mRS 0–1) was similar between the two groups, with no significant differences in the rates of sICH or mortality. TNK appeared associated with shorter IVT-to-groin puncture times [43].

Marnat et al. analyzed data from the ETIS and TETRIS observational registries, focusing on 753 patients with tandem occlusions, of whom 124 received TNK and 629 received ALT prior to EVT. Favorable outcomes, defined as 90-day mRS 0–2, as well as the rates of sICH and parenchymal hematoma, were comparable between groups. However, TNK use was associated with significantly higher rates of final successful recanalization and lower 90-day mortality [44].

In a Spanish study by García-Alcántara et al., 256 patients with LVO were included, with 96 receiving TNK and 160 receiving ALT. Rates of recanalization and ICH were similar between the groups. Functional outcomes were also comparable, regardless of thrombus migration status. Notably, thrombus migration occurred more frequently in patients treated with TNK [45].

#### 3.3.4. Review, Opinion

A review by Campbell emphasizes the simplicity of TNK bolus administration, ensuring easier transfer between or within hospitals for EVT. Additionally, the author mentions that the benefit of TNK over ALT may be greater in patients with LVO. Hence, TNK seems especially promising in patients with LVO while arranging transport for possible EVT. The author highlights the convenience of TNK in patients with LVO-AIS who cannot access EVT, which is, unfortunately, the majority globally. Even in developed countries, most patients first present to a primary stroke center, without access to EVT, and require interhospital transport to a comprehensive stroke center, with no guarantee of still being eligible for EVT upon arrival [35].

### 3.4. Clinical Scenario 2: Summary of Findings

In one out of four studies, all being sub-studies of AcT RCT, TNK was superior to ALT in achieving excellent functional outcomes, whereas in terms of sICH frequency and mortality, TNK was comparable to ALT [17]. In two studies, TNK was superior to ALT in achieving excellent reperfusion, although this did not translate into better functional outcomes [18,37]. In one study, the superiority of TNK over ALT was found only in patients in whom recanalization was achieved using the aspiration technique, and not stentriever devices [37]. In both included meta-analyses, TNK was associated with improved early recanalization rate up to 24 h from LKW, with one study showing lower mortality with TNK compared with ALT [38]. In either study, there was no difference in sICH occurrence and functional outcomes. In all six included RWSs/CSs, TNK was non-inferior to ALT in terms of functional outcome and sICH frequency. In one study, TNK was associated with higher recanalization rates and lower mortality, as well as shorter IVT-to-groin puncture times [43]. In another study, thrombus migration was more frequent with TNK, albeit without deterioration of functional outcomes [43]. TNK and ALT showed similar effectiveness and safety in hyperglycemic patients that fared worse compared with non-hyperglycemic patients, regardless of IVT medication [41]. A review stresses out the benefit of TNK over ALT, especially in patients with LVO prior to EVT, with a possible facilitatory effect of active thrombolytic on EVT success [35]. The summary of the findings for this clinical scenario is presented in Table 2.

### 3.5. Clinical Scenario 3: Tenecteplase vs. Alteplase for Intra-Arterial (Adjuvant) Thrombolysis Following Endovascular Treatment

Campbell pointed out that there are ongoing studies with adjuvant thrombolytics, without mentioning TNK. Among the papers published within the search period, not a single one addressed this relevant clinical scenario using TNK in IAT [35].

## 4. Discussion

According to our analysis of RCT and RWS/CS data during the study period, TNK seems to be non-inferior to ALT in IVT-only patients with AIS. However, meta-analyses encompassing more than 120,000 patients in total suggest TNK to be superior to ALT in these patients in terms of functional outcomes. Moreover, TNK seems to be associated with significantly shorter DNT, principally due to the ease and simplicity of bolus administration, obviating the need for an infusion. In patients with LVO AIS undergoing EVT, prior IVT with TNK seems to be superior to ALT in terms of functional outcomes, shorter DGPT, excellent reperfusion, and improved early recanalization, with a similar safety profile. The benefit of TNK over ALT seems to be higher in patients with LVO AIS compared with non-LVO AIS due to a possible facilitatory effect of active thrombolysis on EVT success.

It is reassuring that TNK showed at least similar results to ALT in a vast population of IVT-only AIS patients, even in the presence of diabetes or hyperglycemia, in elderly patients, in carotid tandem lesions, in posterior circulation occlusion, and in minor AIS. On the contrary, the TEMPO-2 trial, which included patients with minor non-disabling AIS with a proven intracranial occlusion (LVO in 11%, MeVO in 54%), showed that TNK was associated with higher mortality when compared with DAPT in these patients [13]. However, since DAPT, according to international guidelines, is considered as short-term secondary prevention rather than acute treatment in minor AIS patients, these results, in our view, should not prompt clinicians to refrain from IVT in this patient cohort altogether [46]. Instead, taking into account clinical data (e.g., NIHSS, premorbid mRS) as well as radiologic characteristics (e.g., functional imaging), cautious and judicious use of TNK in this population seems reasonable.

On the basis of meta-analyses, TNK holds promise as a potentially preferred treatment option for IVT in AIS, with improved functional outcomes compared with ALT, especially in Caucasians, whereas improved recanalization rates have been observed in Asians [25,26]. A higher frequency of any intracranial hemorrhage, including sICH, without concomitant worsening of mortality or functional outcomes with TNK compared with ALT, was found only in one meta-analysis [22]. This could be associated with a higher recanalization rate with TNK, accompanied by recanalization hemorrhage [47]. A meta-analysis even suggested lower mortality with TNK vs. ALT [23].

Regarding the cost of TNK, it seems to be more cost-efficient than an equivalent ALT dose, especially in some countries, such as China and Slovenia [14]. However, some authors point out that poor waste management in countries where only 50 mg vials of TNK are available could undermine this overall cost-effectiveness [28]. On the contrary, in the authors’ home countries (Slovenia/Germany), where 25 mg vials of TNK are also readily available, waste management can be improved, resulting in considerable cost savings (~20%; unpublished data) compared with ALT.

Regarding workflow in AIS, TNK, due to its bolus-only administration, leads to shorter in-hospital delays in IVT, and significantly shortens DIDO times in patients with LVO who are transferred from a primary stroke center to a comprehensive stroke center for EVT [4,33]. In our view, TNK offers advantages during sedation, requiring only one line. Further, bolus-only administration of TNK assures that a full dose is given to every patient, without the frequent non-trivial interruptions in the administration of ALT that occur in daily clinical practice because of logistical reasons.

Although both TNK and ALT are only approved for use within the 4.5 h time window, and guidelines so far only recommend ALT for up to 9 h using perfusion imaging, Campbell predicts that the time window for TNK might soon be extended up to 24 h from LKW using perfusion-imaging selection [35,46]. In these patients, it has been convincingly shown that the use of TNK has at least not led to worse neurological outcomes [48]. Consequently, TNK is being adopted into international stroke guidelines, with a reasonable expectation that it may fully replace ALT in the future for IVT in all AIS patients [49].

An important issue regarding the interpretation of TNK data is the use of different TNK doses (0.25 mg/kg vs. 0.40 mg/kg), resulting in safety concerns. The European Stroke Organisation (ESO) guidelines suggest that there is sufficient data to support the exclusive use of the 0.25 mg/kg dose of TNK in AIS [49]. Furthermore, these guidelines recommend strongly against the use of the higher 0.40 mg/kg dose, due to the lack of additional benefits as shown by Huang et al. [21], and numerically higher sICH rates associated with high-dose administration [49].

Previous studies showed improved efficacy and safety of TNK, especially in terms of recanalization/reperfusion, in patients with LVO AIS, particularly as a bridging therapy [7,50,51,52]. In the four included sub-analyses of the Canadian AcT trial, TNK was superior to ALT in achieving excellent reperfusion, which, however, did not translate into improved functional outcomes [17,18,37]. Interestingly, in contrast to stentriever devices for EVT, only the aspiration technique led to improved results with TNK compared with ALT [37]. This could be explained by a reduction in fibrin proportion and thrombus volume in TNK-treated patients, which makes it less adherent to the vessel wall [53]. It is known that contact aspiration is less effective with fibrin-rich thrombi, hence TNK may lead to greater reperfusion rates compared with ALT. This effect may be less relevant for stentriever devices [54].

Improved early recanalization rate with TNK has been shown in two meta-analyses, translating into lower mortality in one study [38]. Importantly, the advantages of bolus-only administration of TNK over ALT become more pronounced in patients with LVO AIS, with shorter DGPT [43]. Even though thrombus migration is probably more frequent with TNK compared with ALT, functional outcomes appear comparable [45].

Our literature search did not yield any studies concerning TNK as an adjunct to IAT after EVT, despite this being a very important topic for improving outcomes in these patients. The ANGEL-TNK trial demonstrated that patients with anterior LVO presenting 4.5 to 24 h from symptom onset may benefit from IAT with TNK following successful recanalization, without increased bleeding or mortality risks [55]. These results support the role of IAT in combination with ALT (CHOICE and PEARL) studies.

Two additional considerations regarding TNK warrant further investigation. The first involves patients taking dabigatran; while the ESO guidelines recommend ALT for patients with AIS who have recently taken dabigatran and received idarucizumab, TNK is not explicitly endorsed in this setting. However, emerging evidence indicates that it may be safely used following idarucizumab reversal [46,56]. In line with this, drug approvals stipulate that patients on dabigatran regain eligibility for IVT after administration of idarucizumab, regardless of the use of ALT or TNK. Nevertheless, additional studies and interim guidance are needed to support its use in this subgroup. The second consideration concerns patients with CRAO. Although current guidelines recommend ALT for this indication, some CS have described the use of TNK [57,58]. Nonetheless, more robust clinical trials are necessary to clearly define its efficacy and safety in this context.

Our analysis has clear limitations, most notably its narrative review design. However, its principal strength lies in the comprehensive inclusion of all available data on IVT and EVT from the study period.

## 5. Conclusions

TNK seems to be effective and safe in a wide patient population of IVT-only AIS patients and as a bridging therapy in EVT patients. Its ease of bolus-only administration enables shorter DNT, simplifies, and most importantly, shortens delays in patients with LVO who are candidates for EVT. We propose ongoing collection of real-life data on TNK use in daily clinical practice, additional RCTs, including TNK in IAT, and, importantly, international collaborative efforts. Our narrative review demonstrates the safety and efficacy of TNK as an alternative to ALT in patients with AIS who are candidates for IVT. Owing to its favorable characteristics—including simplified administration, greater fibrin specificity, and improved cost-efficiency, at least in some countries, TNK seems to be a promising option for thrombolytic therapy.

## Figures and Tables

**Table 1:** Summary of findings for clinical scenario 1: tenecteplase vs. alteplase in intravenous thrombolysis-only patients.

Type of Study	Nr. of Included Studies	Main Findings
RCTs	10	Comparable outcomes across subgroups (elderly, diabetes, posterior circulation, tandem carotid lesions, minor AIS) Non-disabling minor AIS + LVO → ↑ mortality vs. DAPT Lower cost (~30% in China)
Meta-analyses	7	2/7: Better functional outcomes (esp. Caucasians), ↑ recanalization in Asians 1/7: ↑ sICH and asymptomatic ICH (no ↑ mortality) 1/7: ↓ mortality
Real-world studies	6	No differences in mortality or sICH Cost savings (but may be offset by waste issues) Shorter DNT, no outcome change
Reviews	3	2/3: superior for excellent outcomes, guideline inclusion predicted Bolus simplicity ensures full dosing; benefit in LVO Suggested window extension up to 24 h with perfusion imaging

RCTs—randomized controlled trials, AIS—acute ischemic stroke, LVO—large vessel occlusion, DAPT—dual antiplatelet therapy, sICH—symptomatic intracranial hemorrhage, ICH—intracranial hemorrhage, DNT—door-to-needle time, →—leads to, ↑—higher, ↓—lower.

**Table 2:** Summary of findings for clinical scenario 2: tenecteplase vs. alteplase as bridging therapy prior to endovascular therapy.

Type of Study	Nr. of Included Studies	Main Findings
AcT sub-studies	4	1/4: superior for excellent outcomes sICH and mortality were comparable
Meta-analyses	2	Improved early recanalization ≤24 h from LKW 1/2: ↓ mortality No differences in sICH or functional outcomes
Real-world studies	6	Non-inferior for outcomes and sICH 1/6: ↑ recanalization, ↓ mortality, shorter IVT-to-groin-puncture time 1/6: ↑ thrombus migration (no worse outcomes) Hyperglycemia: TNK ≈ ALT, both worse vs. normoglycemia
Reviews	1	May benefit LVO-AIS before EVT Facilitatory effect of active IVT on EVT success

AcT—intravenous tenecteplase compared with alteplase for acute ischemic stroke in Canada, sICH—symptomatic intracranial hemorrhage, LKW—last known well, IVT—intravenous thrombolysis, TNK—tenecteplase, ALT—alteplase, LVO—large vessel occlusion, AIS—acute ischemic stroke, EVT—endovascular treatment, ↑—higher, ↓—lower.

## Data Availability

The dataset is available on request from the authors.

## Abbreviations

The following abbreviations are used in this manuscript:

TNK	Tenecteplase
ALT	Alteplase
IVT	Intravenous thrombolysis
AIS	Acute ischemic stroke
EVT	Endovascular treatment
rt-PA	Recombinant tissue plasminogen activator
DNT	Door-to-needle time
DGPT	Door-to-groin puncture time
DIDO	Door-in-door-out time
RCTs	Randomized controlled trials
LKW	Last known well
CRAO	Central retinal artery occlusion
IAT	Intra-arterial thrombolysis
sICH	Symptomatic intracranial hemorrhage
ESO	European Stroke Organisation
RWS	Real-world studies
CS	Case series
mRS	Modified Rankin Score
NIHSS	National Institute of Health Stroke Scale
LVO	Large vessel occlusion
MeVO	Medium vessel occlusion
DAPT	Dual antiplatelet therapy
ICA	Internal carotid artery
MCA	Middle cerebral artery
TICI	Thrombolysis in cerebral infarction

## References

[1] Emberson J., Lees K.R., Lyden P., Blackwell L., Albers G., Bluhmki E., Brott T., Cohen G., Davis S., Donnan G. (2014). Effect of Treatment Delay, Age, and Stroke Severity on the Effects of Intravenous Thrombolysis with Alteplase for Acute Ischaemic Stroke: A Meta-Analysis of Individual Patient Data from Randomised Trials. Lancet.

[2] Campbell B.C.V., Ma H., Ringleb P.A., Parsons M.W., Churilov L., Bendszus M., Levi C.R., Hsu C., Kleinig T.J., Fatar M. (2019). Extending Thrombolysis to 4.5–9 h and Wake-up Stroke Using Perfusion Imaging: A Systematic Review and Meta-Analysis of Individual Patient Data. Lancet.

[3] Tanswell P., Modi N., Combs D., Danays T. (2002). Pharmacokinetics and Pharmacodynamics of Tenecteplase in Fibrinolytic Therapy of Acute Myocardial Infarction. Clin. Pharmacokinet..

[4] Warach S.J., Dula A.N., Milling T.J., Miller S., Allen L., Zuck N.D., Miller C., Jesser C.A., Misra L.R., Miley J.T. (2022). Prospective Observational Cohort Study of Tenecteplase Versus Alteplase in Routine Clinical Practice. Stroke.

[5] Zhong C.S., Beharry J., Salazar D., Smith K., Withington S., Campbell B.C.V., Wilson D., Le Heron C., Mason D., Duncan R. (2021). Routine Use of Tenecteplase for Thrombolysis in Acute Ischemic Stroke. Stroke.

[6] Parsons M.W., Yogendrakumar V., Churilov L., Garcia-Esperon C., Campbell B.C.V., Russell M.L., Sharma G., Chen C., Lin L., Chew B.L. (2024). Tenecteplase versus Alteplase for Thrombolysis in Patients Selected by Use of Perfusion Imaging within 4.5 h of Onset of Ischaemic Stroke (TASTE): A Multicentre, Randomised, Controlled, Phase 3 Non-Inferiority Trial. Lancet Neurol..

[7] Campbell B.C.V., Mitchell P.J., Churilov L., Yassi N., Kleinig T.J., Dowling R.J., Yan B., Bush S.J., Dewey H.M., Thijs V. (2018). Tenecteplase versus Alteplase before Thrombectomy for Ischemic Stroke. N. Engl. J. Med..

[8] Bivard A., Zhao H., Churilov L., Campbell B.C.V., Coote S., Yassi N., Yan B., Valente M., Sharobeam A., Balabanski A.H. (2022). Comparison of Tenecteplase with Alteplase for the Early Treatment of Ischaemic Stroke in the Melbourne Mobile Stroke Unit (TASTE-A): A Phase 2, Randomised, Open-Label Trial. Lancet Neurol..

[9] Menon B.K., Buck B.H., Singh N., Deschaintre Y., Almekhlafi M.A., Coutts S.B., Thirunavukkarasu S., Khosravani H., Appireddy R., Moreau F. (2022). Intravenous Tenecteplase Compared with Alteplase for Acute Ischaemic Stroke in Canada (AcT): A Pragmatic, Multicentre, Open-Label, Registry-Linked, Randomised, Controlled, Non-Inferiority Trial. Lancet.

[10] Wang Y., Li S., Pan Y., Li H., Parsons M.W., Campbell B.C.V., Schwamm L.H., Fisher M., Che F., Dai H. (2023). Tenecteplase versus Alteplase in Acute Ischaemic Cerebrovascular Events (TRACE-2): A Phase 3, Multicentre, Open-Label, Randomised Controlled, Non-Inferiority Trial. Lancet.

[11] Palaiodimou L., Katsanos A.H., Turc G., Romoli M., Theodorou A., Lemmens R., Sacco S., Velonakis G., Vlachopoulos C., Tsivgoulis G. (2024). Tenecteplase for the Treatment of Acute Ischemic Stroke in the Extended Time Window: A Systematic Review and Meta-Analysis. Ther. Adv. Neurol. Disord..

[12] Muir K.W., Ford G.A., Ford I., Wardlaw J.M., McConnachie A., Greenlaw N., Mair G., Sprigg N., Price C.I., MacLeod M.J. (2024). Tenecteplase versus Alteplase for Acute Stroke within 4·5 h of Onset (ATTEST-2): A Randomised, Parallel Group, Open-Label Trial. Lancet Neurol..

[13] Coutts S.B., Ankolekar S., Appireddy R., Arenillas J.F., Assis Z., Bailey P., Barber P.A., Bazan R., Buck B.H., Butcher K.S. (2024). Tenecteplase versus Standard of Care for Minor Ischaemic Stroke with Proven Occlusion (TEMPO-2): A Randomised, Open Label, Phase 3 Superiority Trial. Lancet.

[14] Xiong Y., Wang L., Pan Y., Wang M., Schwamm L.H., Duan C., Campbell B.C.V., Li S., Hao M., Wu N. (2025). Tenecteplase versus Alteplase for Acute Ischaemic Stroke in the Elderly Patients: A Post Hoc Analysis of the TRACE-2 Trial. Stroke Vasc. Neurol..

[15] Liu H., Jin A., Pan Y., Meng X., Li H., Li Z., Wang Y., Li S. (2024). Efficacy and Safety of Intravenous Tenecteplase Versus Alteplase in Treating Acute Ischemic Stroke with Diabetes and Admission Hyperglycemia. J. Am. Heart Assoc..

[16] Cimflova P., Alhabli I., Bala F., Horn M., Benali F., Buck B.H., Catanese L., Coutts P.S.B., Khosravani H., Appireddy R. (2024). Intravenous Alteplase versus Tenecteplase in Patients with Acute Posterior Circulation Strokes: A Secondary Analysis from the AcT Randomized Controlled Trial. J. Stroke Cerebrovasc. Dis..

[17] Bala F., Almekhlafi M., Singh N., Alhabli I., Ademola A., Coutts S.B., Deschaintre Y., Khosravani H., Appireddy R., Moreau F. (2024). Safety and Efficacy of Tenecteplase versus Alteplase in Stroke Patients with Carotid Tandem Lesions: Results from the AcT Trial. Int. J. Stroke.

[18] Bala F., Singh N., Ignacio K., Alhabli I., Ademola A., Alrohimi A., Khosravani H., Tkach A., Catanese L., Dowlatshahi D. (2024). Tenecteplase Versus Alteplase in Medium Vessel Occlusion Ischemic Stroke: A Secondary Analysis of the Alteplase Compared to Tenecteplase Randomized Trial. J. Stroke.

[19] Nair R., Singh N., Kate M., Asdaghi N., Sarmiento R., Bala F., Coutts S.B., Horn M., Poppe A.Y., Williams H. (2024). Intravenous Tenecteplase Compared with Alteplase for Minor Ischaemic Stroke: A Secondary Analysis of the AcT Randomised Clinical Trial. Stroke Vasc. Neurol..

[20] Cheng X., Hong L., Lin L., Churilov L., Ling Y., Yang N., Fu J., Lu G., Yue Y., Zhang J. (2025). Tenecteplase Thrombolysis for Stroke up to 24 Hours After Onset with Perfusion Imaging Selection: The CHABLIS-T II Randomized Clinical Trial. Stroke.

[21] Huang J., Zheng H., Zhu X., Zhang K., Ping X. (2024). Tenecteplase versus Alteplase for the Treatment of Acute Ischemic Stroke: A Meta-Analysis of Randomized Controlled Trials. Ann. Med..

[22] Singh A., Singh M.P., Gaikwad N.R., Kannauje P.K. (2024). Tenecteplase versus Alteplase in Acute Ischemic Stroke: A Systematic Review and Meta-Analysis. Ann. Neurosci..

[23] Ma Y., Xiang H., Busse J.W., Yao M., Guo J., Ge L., Li B., Luo X., Mei F., Liu J. (2024). Tenecteplase versus Alteplase for Acute Ischemic Stroke: A Systematic Review and Meta-Analysis of Randomized and Non-Randomized Studies. J. Neurol..

[24] Xiong Y., Wang L., Li G., Yang K.-X., Hao M., Li S., Pan Y., Wang Y. (2024). Tenecteplase versus Alteplase for Acute Ischaemic Stroke: A Meta-Analysis of Phase III Randomised Trials. Stroke Vasc. Neurol..

[25] Wang Y., Cai X., Fang Q., Zhu J. (2024). Efficacy and Safety Outcomes of Tenecteplase versus Alteplase for Thrombolysis of Acute Ischemic Stroke: A Meta-Analysis of 9 Randomized Controlled Trials. J. Neurol. Sci..

[26] Koh J.H., Lim C.Y.J., Tan L.T.P., Sia C.-H., Poh K.K., Sharma V.K., Yeo L.L.L., Ho A.F.W., Wu T., Kong W.K.-F. (2024). Ethnic Differences in the Safety and Efficacy of Tenecteplase Versus Alteplase for Acute Ischemic Stroke: A Systematic Review and Meta-Analysis. J. Stroke.

[27] Günkan A., Ferreira M.Y., Vilardo M., Scarcia L., Bocanegra-Becerra J.E., Cardoso L.J.C., Fabrini Paleare L.F., De Oliveira Almeida G., Semione G., Ferreira C. (2025). Thrombolysis for Ischemic Stroke Beyond the 4.5-Hour Window: A Meta-Analysis of Randomized Clinical Trials. Stroke.

[28] Cassano C., Schiller D., Fulman M. (2025). Navigating the Shift: Comparing Safety and Cost of Tenecteplase versus Alteplase in Acute Ischemic Stroke. Neurohospitalist.

[29] Lo W.T., Fong W.C., Chau C.S.K., Ismail M., Li J.T.C., Chan C.C., Chan C.H.S., Chan C.Y., Chan G.H.-F., Chan A.L.-T. (2024). Safety and Efficacy Comparison of Tenecteplase and Alteplase for Clinically Suspected Large Vessel Occlusion Strokes without Thrombectomy. Cerebrovasc. Dis. Extra.

[30] Dutta A., Gupta S., Chakraborty U., Mondal C., Banerjee S., Das D., Jatua S.K., Chakrabarty S., Misra S., Bhattacharya J. (2024). Comparative Analysis of Tenecteplase versus Alteplase in Acute Ischemic Stroke: A Multicentric Observational Study from Eastern India. Ann. Indian Acad. Neurol..

[31] Guzman M., Lavados P.M., Cavada G., Brunser A.M., Olavarria V.V. (2025). Emergency Department Workflow Times of Intravenous Thrombolysis with Tenecteplase versus Alteplase in Acute Ischemic Stroke: A Prospective Cohort Study before and during the COVID-19 Pandemic. Cerebrovasc. Dis. Extra.

[32] Skärlund M., Åsberg S., Eriksson M., Lundström E. (2024). Tenecteplase Compared to Alteplase in Real-World Outcome: A Swedish Stroke Register Study. Upsala J. Med. Sci..

[33] Henderson B., Emborski R., Diioia A., Stone D., Stupca K. (2024). Improved Door-to-Needle Time After Implementation of Tenecteplase as the Preferred Thrombolytic for Acute Ischemic Stroke at a Large Community Teaching Hospital Emergency Department. Hosp. Pharm..

[34] Toyoda K. (2024). Tenecteplase versus Alteplase in Stroke Thrombolysis: The Last Piece of the Puzzle?. Lancet Neurol..

[35] Campbell B.C. (2024). Hyperacute Ischemic Stroke Care—Current Treatment and Future Directions. Int. J. Stroke.

[36] Wang X., Dong Y., Dong Q., Wang D. (2024). Should Patients with Minor Strokes Be given Thrombolytics?. Stroke Vasc. Neurol..

[37] Bala F., Diprose W., Menon B.K., Singh N., Khosravani H., Tkach A., Catanese L., Dowlatshahi D., Field T.S., Hunter G. (2025). Effect of Thrombolysis Type on the Efficacy of Aspiration versus Stent Retriever First Line Thrombectomy: Results from the AcT Trial. J. NeuroIntervent. Surg..

[38] Wu N., Doeppner T.R., Hermann D.M., Gronewold J. (2024). Efficacy and Safety of Intravenous Tenecteplase Compared to Alteplase before Mechanical Thrombectomy in Acute Ischemic Stroke: A Meta-Analysis. J. Neurol..

[39] Guo S., Qin S., Tan S., Su H., Chen X. (2024). Endovascular Thrombectomy without versus with Different Pre-Intravenous Thrombolysis in Acute Ischemic Stroke: A Network Meta-Analysis of Randomized Controlled Trials. Front. Neurol..

[40] Hendrix P., Gross B.A., Allahdadian S., Sioutas G.S., Koul P., Tarbay A.C., Lang M.J., Srinivasan V.M., Al-Bayati A.R., Li J. (2024). Tenecteplase versus Alteplase before Stroke Thrombectomy: Outcomes after System-Wide Transitions in Pennsylvania. J. Neurol..

[41] Hendrix P., Koul P., Noto A., Li J., Schirmer C.M., Lang M.J., Al-Bayati A.R., Nogueira R.G., Gross B.A. (2024). Admission Hyperglycemia Effect on Symptomatic Intracranial Hemorrhage in Tenecteplase versus Alteplase before Large Vessel Occlusion Stroke Thrombectomy. J. Neurol..

[42] Karamchandani R.R., Asimos A.W., Strong D., Rhoten J.B., Clemente J.D., Defilipp G., Bernard J.D., Stetler W.R., Parish J.M., Hines A.U. (2024). Early Recanalization after Tenecteplase versus Alteplase: Experience in a Large Stroke Network. J. Stroke Cerebrovasc. Dis..

[43] Zarzour A., Batot C., Boisseau W., Cho T.-H., Guillon B., Richard S., Marnat G., Arquizan C., Lapergue B., Weisenburger Lile D. (2024). Tenecteplase versus Alteplase before Thrombectomy: A Comprehensive Evaluation of Clinical and Angiographic Impact: Insights from the ETIS Registry. J. Neuroradiol..

[44] Marnat G., Lapergue B., Gory B., Kyheng M., Labreuche J., Turc G., Olindo S., Sibon I., Caroff J., Smadja D. (2024). Intravenous Thrombolysis with Tenecteplase versus Alteplase Combined with Endovascular Treatment of Anterior Circulation Tandem Occlusions: A Pooled Analysis of ETIS and TETRIS. Eur. Stroke J..

[45] García-Alcántara G., Moreno-López C., López-Rebolledo R., Lorenzo-Barreto P., Garay-Albízuri P., Martínez-García B., Llanes A., Pérez-Gil D., Chico J.L., Vera-Lechuga R. (2025). Clot Migration in Patients Treated with Tenecteplase versus Alteplase before Mechanical Thrombectomy. Eur. Stroke J..

[46] Berge E., Whiteley W., Audebert H., De Marchis G., Fonseca A.C., Padiglioni C., Pérez De La Ossa N., Strbian D., Tsivgoulis G., Turc G. (2021). European Stroke Organisation (ESO) Guidelines on Intravenous Thrombolysis for Acute Ischaemic Stroke. Eur. Stroke J..

[47] Nie X., Pu Y., Zhang Z., Liu X., Duan W., Liu L. (2018). Futile Recanalization after Endovascular Therapy in Acute Ischemic Stroke. BioMed Res. Int..

[48] Albers G.W., Jumaa M., Purdon B., Zaidi S.F., Streib C., Shuaib A., Sangha N., Kim M., Froehler M.T., Schwartz N.E. (2024). Tenecteplase for Stroke at 4.5 to 24 Hours with Perfusion-Imaging Selection. N. Engl. J. Med..

[49] Alamowitch S., Turc G., Palaiodimou L., Bivard A., Cameron A., De Marchis G.M., Fromm A., Kõrv J., Roaldsen M.B., Katsanos A.H. (2023). European Stroke Organisation (ESO) Expedited Recommendation on Tenecteplase for Acute Ischaemic Stroke. Eur. Stroke J..

[50] Parsons M., Spratt N., Bivard A., Campbell B., Chung K., Miteff F., O’Brien B., Bladin C., McElduff P., Allen C. (2012). A Randomized Trial of Tenecteplase versus Alteplase for Acute Ischemic Stroke. N. Engl. J. Med..

[51] Kheiri B., Osman M., Abdalla A., Haykal T., Ahmed S., Hassan M., Bachuwa G., Al Qasmi M., Bhatt D.L. (2018). Tenecteplase versus Alteplase for Management of Acute Ischemic Stroke: A Pairwise and Network Meta-Analysis of Randomized Clinical Trials. J. Thromb. Thrombolysis.

[52] Bivard A., Huang X., Levi C.R., Spratt N., Campbell B.C.V., Cheripelli B.K., Kalladka D., Moreton F.C., Ford I., Bladin C.F. (2017). Tenecteplase in Ischemic Stroke Offers Improved Recanalization: Analysis of 2 Trials. Neurology.

[53] Frühwald T., Gärtner U., Stöckmann N., Marxsen J.-H., Gramsch C., Roessler F.C. (2019). In Vitro Examination of the Thrombolytic Efficacy of Tenecteplase and Therapeutic Ultrasound Compared to Rt-PA. BMC Neurol..

[54] Bala F., Kappelhof M., Ospel J.M., Cimflova P., Qiu W., Singh N., Zhu K., Kim B.J., Wadhwa A., Almekhlafi M.A. (2023). Distal Embolization in Relation to Radiological Thrombus Characteristics, Treatment Details, and Functional Outcome. Stroke.

[55] ISC 2025 Session Report: Intra-Arterial Tenecteplase After Successful Recanalization Improves Neurological Outcomes. https://www.ahajournals.org/do/10.1161/blog.20250304.837897.

[56] Beharry J., Waters M.J., Drew R., Fink J.N., Wilson D., Campbell B.C.V., Parsons M.W., Kleinig T.J., Wu T.Y. (2020). Dabigatran Reversal Before Intravenous Tenecteplase in Acute Ischemic Stroke. Stroke.

[57] Mac Grory B., Schrag M., Biousse V., Furie K.L., Gerhard-Herman M., Lavin P.J., Sobrin L., Tjoumakaris S.I., Weyand C.M., Yaghi S. (2021). Management of Central Retinal Artery Occlusion: A Scientific Statement From the American Heart Association. Stroke.

[58] Vo A., Hicks W., Sangha N. (2024). A Case Series on Treatment of Central and Branch Retinal Artery Occlusion with Intravenous Tenecteplase. J. Stroke Cerebrovasc. Dis..

